# Characterization of the *Nrt2.6* Gene in *Arabidopsis thaliana*: A Link with Plant Response to Biotic and Abiotic Stress

**DOI:** 10.1371/journal.pone.0042491

**Published:** 2012-08-07

**Authors:** Julie Dechorgnat, Oriane Patrit, Anne Krapp, Mathilde Fagard, Françoise Daniel-Vedele

**Affiliations:** 1 Institut National de la Recherche Agronomique, Institut Jean-Pierre Bourgin, UMR 1318 INRA-AgroParisTech, Saclay Plant Sciences, Versailles, France; 2 AgroParisTech, UMR217, Laboratoire des Interactions Plantes Pathogènes, Paris, France; 3 Institut National de la Recherche Agronomique, UMR217, Laboratoire des Interactions Plantes Pathogènes, Paris, France; National Taiwan University, Taiwan

## Abstract

The high affinity nitrate transport system in *Arabidopsis thaliana* involves one gene and potentially seven genes from the NRT1 and NRT2 family, respectively. Among them, NRT2.1, NRT2.2, NRT2.4 and NRT2.7 proteins have been shown to transport nitrate and are localized on the plasmalemma or the tonoplast membranes. NRT2.1, NRT2.2 and NRT2.4 play a role in nitrate uptake from soil solution by root cells while NRT2.7 is responsible for nitrate loading in the seed vacuole. We have undertaken the functional characterization of a third member of the family, the *NRT2.6* gene. *NRT2.6* was weakly expressed in most plant organs and its expression was higher in vegetative organs than in reproductive organs. Contrary to other NRT2 members, *NRT2.6* expression was not induced by limiting but rather by high nitrogen levels, and no nitrate-related phenotype was found in the *nrt2.6-1* mutant. Consistently, the over-expression of the gene failed to complement the nitrate uptake defect of an *nrt2.1-nrt2.2* double mutant. The *NRT2.6* expression is induced after inoculation of *Arabidopsis thaliana* by the phytopathogenic bacterium *Erwinia amylovora*. Interestingly, plants with a decreased *NRT2.6* expression showed a lower tolerance to pathogen attack. A correlation was found between *NRT2.6* expression and ROS species accumulation in response to infection by *E. amylovora* and treatment with the redox-active herbicide methyl viologen, suggesting a probable link between NRT2.6 activity and the production of ROS in response to biotic and abiotic stress.

## Introduction

Nitrate uptake and nitrate distribution through the whole plant has been intensively studied during the last decade, particularly in the model plant *Arabidopsis thaliana* [1], [2]. Physiological studies have led to the separation of the uptake process into two systems: the high affinity transport system (HATS) and the low affinity transport system (LATS), operating at low (<1 m) or high (>1 mM) external nitrate concentrations, respectively [3]. The molecular organization of the uptake is however much more complex. Indeed, each system combines components inducible or non-inducible by nitrate and each component in turn is encoded by several genes belonging to multigenic families.

The NRT2 family includes 7 members in Arabidopsis that encode potential transporters with high affinity for nitrate. Of these 7 genes, *NRT2.1* and *NRT2.2* are mainly expressed in the root and participate in the high affinity influx of nitrate from soil into root cells [4]. Reverse genetics studies have shown the importance of *NRT2.1* whereas *NRT2.2* activity is only noticeable in the absence of *NRT2.1* [5]. Their expression is strongly induced by low concentrations of nitrate [6] and at least *NRT2.1* is also positively regulated by photosynthesis products [7]. On the other hand, nitrate uptake and *NRT2.1* expression are severely inhibited when reduced nitrogen sources are provided such as ammonium or glutamine [8], [9]. The NRT2.1 protein is localized on the plasma membrane [10], [11]. The NRT2.4 protein is also localized on the plasma membrane and is thought to play a role in nitrate transport activity in the very high affinity range in both roots and shoots under N starvation [12]. In contrast to these transporters, the *NRT2.7* gene is expressed very specifically in the seed, showing a peak of expression during later stages of seed maturation. The protein is localized on the tonoplast and seems to be responsible for the accumulation of nitrate in the vacuoles of seeds [13]. Currently, there is no functional data on other genes of the NRT2 family, *NRT2.3–5–6*. Expression analysis showed that the *NRT2.5* gene is regulated in an opposite way to that of *NRT2.1*: as *NRT2.4*, it is expressed in the absence of nitrate and repressed by an additional exogenous nitrogen source, either as nitrate or ammonium [14]. This kind of regulation suggests that it could play a role in nitrate retrieving in response to limiting nitrogen supply. Indeed, the *NRT1.7* gene coding for a low-affinity nitrate transporter, is positively regulated by nitrogen starvation and null mutants showed growth retardation under starvation [15]. In contrast, the expression of the *NRT2.3* and *NRT2.6* is not affected in response to either nitrogen starvation or nitrogen re-supply, in roots and shoots [14].

Once entered the plant cell, nitrate is directed towards the vacuole to be stored or reduced into nitrite by the cytosolic enzyme nitrate reductase (NR). Then nitrite is translocated to the chloroplast where it is reduced into ammonium by the nitrite reductase (NiR), ammonium which is further incorporated into amino acids by the glutamine synthetase/glutamine synthase cycle. The link between nitrogen assimilation and plant response to microorganisms has been shown in symbiotic as well as in pathogenic interactions [16]. High concentrations of nitrogen often increase susceptibility of plants to disease and even the form of nitrogen available to plants and pathogens can affect the severity of the disease [17]. At the molecular level, bacterial and fungal genes that are induced *in planta* during infection are also induced *in vitro* under nitrogen limiting conditions [18]. Among the complex defense mechanisms set up by plants in response to pathogen attacks [19], one major gene of the nitrate assimilation pathway has been shown to play also a key role in plant-pathogen interactions. The *nia1 nia2* double mutant of Arabidopsis presents an impaired response to an avirulent strain of the bacteria *Pseudomonas syringae* [20]. The nitrate reductase (NR) enzyme, coded by two *Nia* genes in Arabidopsis was thought to produce a key signaling molecule, nitric oxyde (NO), through its associated nitrite-reducing activity [21]. However, it was further demonstrated that the NR activity was not essential for NO synthesis but as an important source of nitrite, through nitrate reduction, for subsequent NO production and plant resistance [20]. Another N-metabolite modified in the double mutant, the L-arginine, can also be used as endogenous substrate for NO synthesis but Oliveira and co-workers [22] showed that the susceptibility of *nia1 nia2* double mutant to this *Pseudomonas* strain did not result from a deficiency in amino acid content. Recently, the nitrate transporter NRT2-1 was also shown to play a role in the resistance against pathogens, linking further nitrate metabolism and plant resistance to biotic stress. Indeed, an *nrt2-1* null mutant was found to be less sensitive to a virulent strain of *P. syringae* pv. tomato [23].

Additionally to its induction after infection by the tumorigenic *Agrobacterium tumefaciens* infection [24], *NRT2.6* mRNAs accumulate also in response to interactions with a plant growth-promoting rhizobacterium (PGPR), which triggers beneficial effects both on plant growth and health [25]. A third bacterium, *Erwinia amylovora*, was shown to have an effect on the *NRT2.6* expression after inoculation in Arabidopsis leaves (CATdb database, urgv.evry.inra.fr/cgi-bin/projects/CATdb/catdb_index.pl). All these results prompted us to characterize the function of *NRT2.6* as a nitrate transporter and its role in the plant, particularly in response to bacterial pathogens. We thus analyze precisely the expression profile of *NRT2.6* and show that it is unable to complement a mutant affected in nitrate transport. However, we uncover an important role of the gene in plant response to pathogen attacks, possibly through the accumulation of reactive oxygen species (ROS).

## Results

### The *NRT2.6* Gene is Weakly Expressed in Many Organs

**Figure 1:**
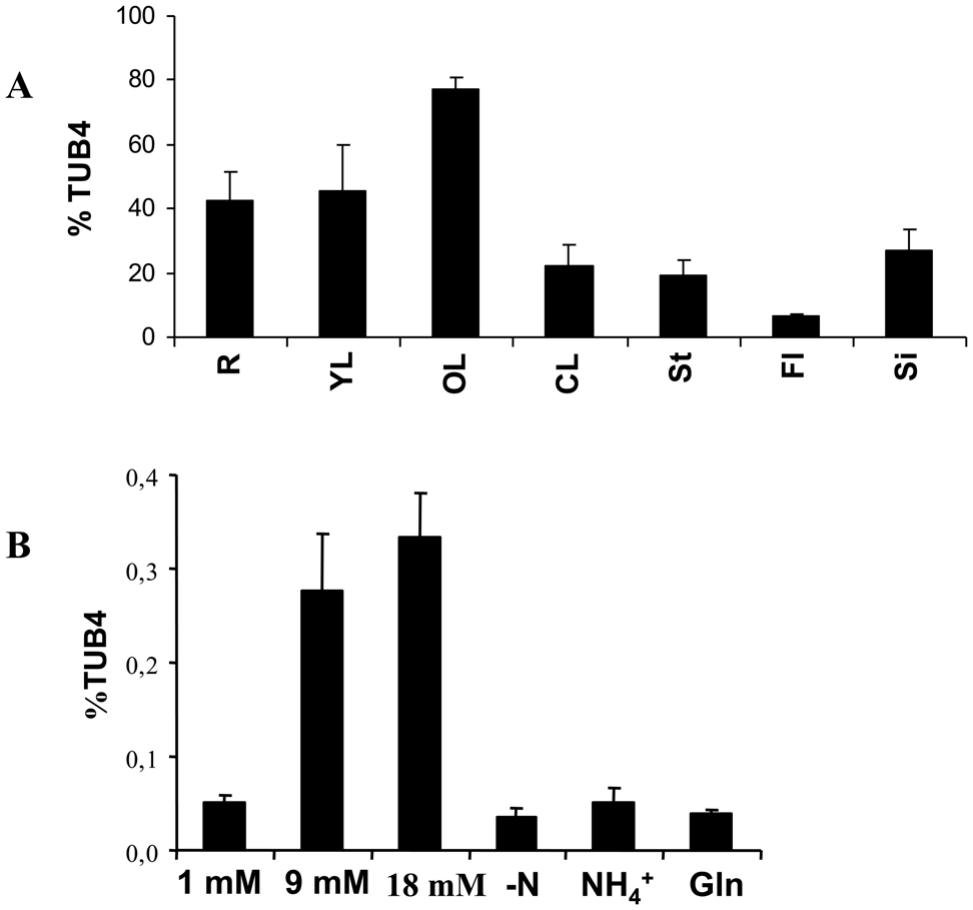
Transcriptional regulation of *NRT2.6* by nitrogen sources. **A**: *NRT2.6* expression in 37 day-old Columbia plants grown in greenhouse on sand with 10 mM nitrate as sole nitrogen source (R: Roots, YL: Young leaves, OL: Old leaves, CL: Cauline leaves, St: Stem, Fl: Flowers, Si: Siliques). The values are means ±SD of three independent plants. **B** *: NRT2.6* expression in plantlets grown on culture medium containing 1, 9 or 18 mM NO_3_^−^, 5 mM NH_4_^+^ or 5 mM glutamine (Gln) as sole nitrogen source or without nitrogen (−N). The values are means ±SD of 4 to 6 independent pools of plantlets.

**Figure 2:**
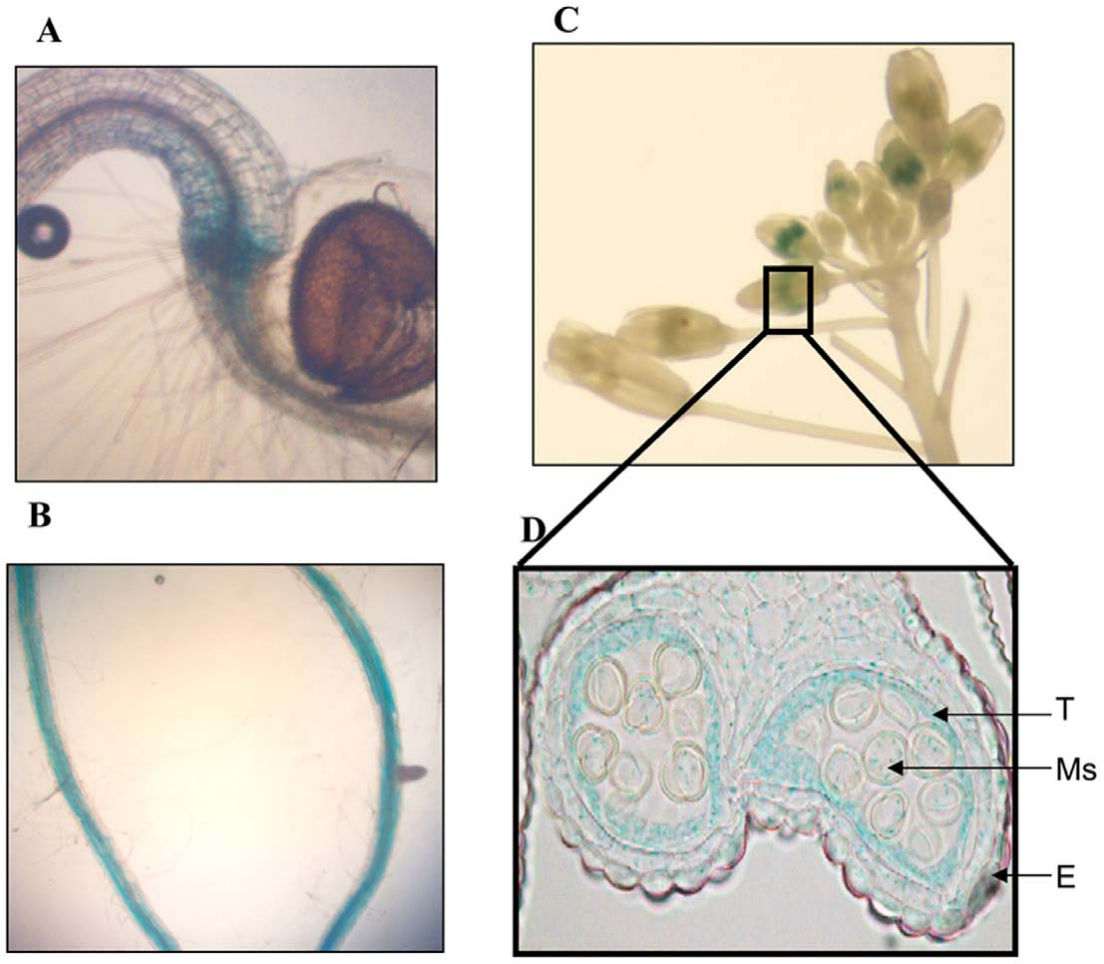
Transcriptional regulation of *ProNRT2.6::uidA* in different plant tissues. **A**: GUS staining of 7 day-old plantlets gown in vitro on culture medium with 9 mM NO_3_^−^. **B**: GUS staining of lateral roots of 25 day-old plants grown in greenhouse with 10 mM NO_3_^−^. **C**: GUS staining in inflorescences of plants grown in greenhouse with 10 mM NO_3_^−^. **D**: Longitudinal cross section of anther : T: tapetum, Ms: meiocytes, E: epidermis.

*NRT2.6* expression profile was detected by quantitative RT-PCR (Figure 1A). Plants were grown in the green house and fed with a standard nutrient solution [26]. The gene is weakly expressed in all plant organs but its expression seems to be slightly higher in vegetative parts (roots and rosette leaves) than in reproductive parts (cauline leaves, stems, siliques and flowers). To confirm these results, we transformed plants with a construct containing the *uidA* reporter gene under the control of *NRT2.6* promoter (Figure 2). Despite the weak expression found by RT-qPCR in all organs, the blue coloration is readily detectable only in lateral roots (Figure 2B) and in the collar of young plantlets, the region between radicle and hypocotyl where roots hair grow (Figure 2A). The gene is also expressed in inflorescences but the level of *uidA* expression is dependent on the anther developing stage (Figure 2C). To investigate more precisely the cellular localization of *NRT2.6* mRNAs, we performed histochemical studies of anthers and found that the blue coloration is very specific of the specialized tapetal cells (Figure 2D). This layer surrounding the locule is transient and allows pollen development [27]. This highly specific localization led us to investigate the potential effect of a mutation in the *NRT2.6* gene on the transmission of male gametophyte to the progeny and pollen viability. However, no difference concerning these two parameters was detected between a null mutant (see below) and the wild-type (Figure S1).

In order to investigate the effect of nitrogen sources on gene expression, we grew plantlets during 4 days on different culture media containing nitrate, ammonium or glutamine as sole nitrogen sources or without nitrogen (Figure 1B). The gene expression was significantly lower (100 times less) in *in vitro* grown young plantlets compared to sand conditions (Figure 1, A and B). The expression of *NRT2.6* was weak and similar in the absence of nitrogen and in low nitrate condition (1 mM). This expression increased as nitrate availability increased but the expression level was the same whether in full or very high nitrate supply (9 or 18 mM, respectively). When the nitrogen source was provided by ammonium or glutamine the *NRT2.6* mRNAs levels were similar to those in low nitrate (1 mM) or in the absence of nitrogen. Thus, *NRT2.6* expression seems to be induced by high levels of nitrate supply but, in contrast to other NRT2 members, is not sensitive to reduced nitrogen forms.

### Is the NRT2.6 Protein Able to Transport Nitrate?

As NRT2.6 shares 67% of homology with the NRT2.1 protein [28], we asked the question of whether or not a deficiency in the NRT2.6 could have also an effect on nitrate uptake. Among all the T-DNA collections, only one T-DNA insertion line was available. The *nrt2.6-1* mutant in Columbia accession was obtained from the NASC center (NASC ID: N121890). The mutant was isolated from an insertional mutagenesis based on the maize En/Spm element [29]. In this mutant, one T-DNA copy was inserted in the beginning of the second exon (Figure S2). No expression of full-size cDNA was detected by RT-PCR (data not shown), demonstrating that it is a null mutant.

**Figure 3:**
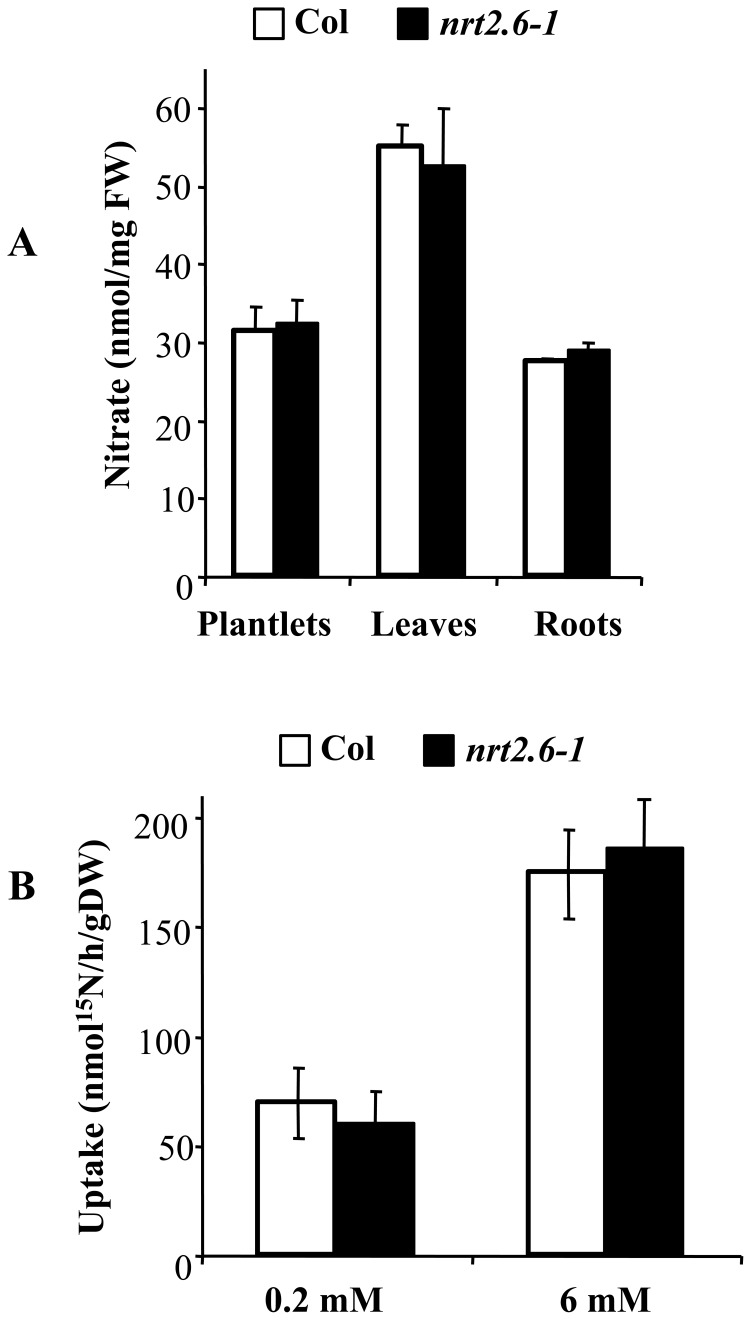
Nitrate contents and root nitrate influx in Col and *nrt2.6-1* mutant. **A**: Nitrate contents of plants were grown either in vitro with 9 mM NO_3_^−^ (plantlets) or on sand with 10 mM NO_3_^−^ (leaves and roots). The values are means ±SD of 5 to 6 plants. **B**: Nitrate influx of plant roots. Plants were grown in hydroponic culture on 0.5 mM NH_4_NO_3_ for 42 days and then transferred for 1 additional week to 0.2 mM NO_3_^−^. Root influx was measured at both 0.2 and 6 mM ^15^NO_3_^−^ to provide estimation of HATS and HATS + LATS activities, respectively. The values are means ±SD of 5 to 6 plants.

We first measured the nitrate contents in both genotypes. As shown in Figure 3A, the mutant accumulates the same nitrate contents than the wild-type whether it is grown *in vitro* with 9 mM nitrate as the sole nitrogen source or on sand in the greenhouse with 10 mM nitrate (see Materials and Methods). We then measured the nitrate uptake capacity of the *nrt2.6-1* mutant. Plants were grown under hydroponic culture conditions during 35 short days and fed with 0.5 mM NH_4_NO_3_ and then 7 days with 0.2 mM NO_3_^−^ before the uptake experiment. Two concentrations of ^15^N were used: 0.2 and 6 mM ^15^NO_3_^−^ to measure nitrate uptake mediated by HATS and HATS + LATS, respectively. The *nrt2.6-1* mutant has exactly the same uptake as the Columbia wild-type genotype, whatever the concentration of ^15^NO_3_^−^ (Figure 3B). Thus, the loss of NRT2.6 does not seem to modify NO_3_^−^ import by roots.

**Figure 4:**
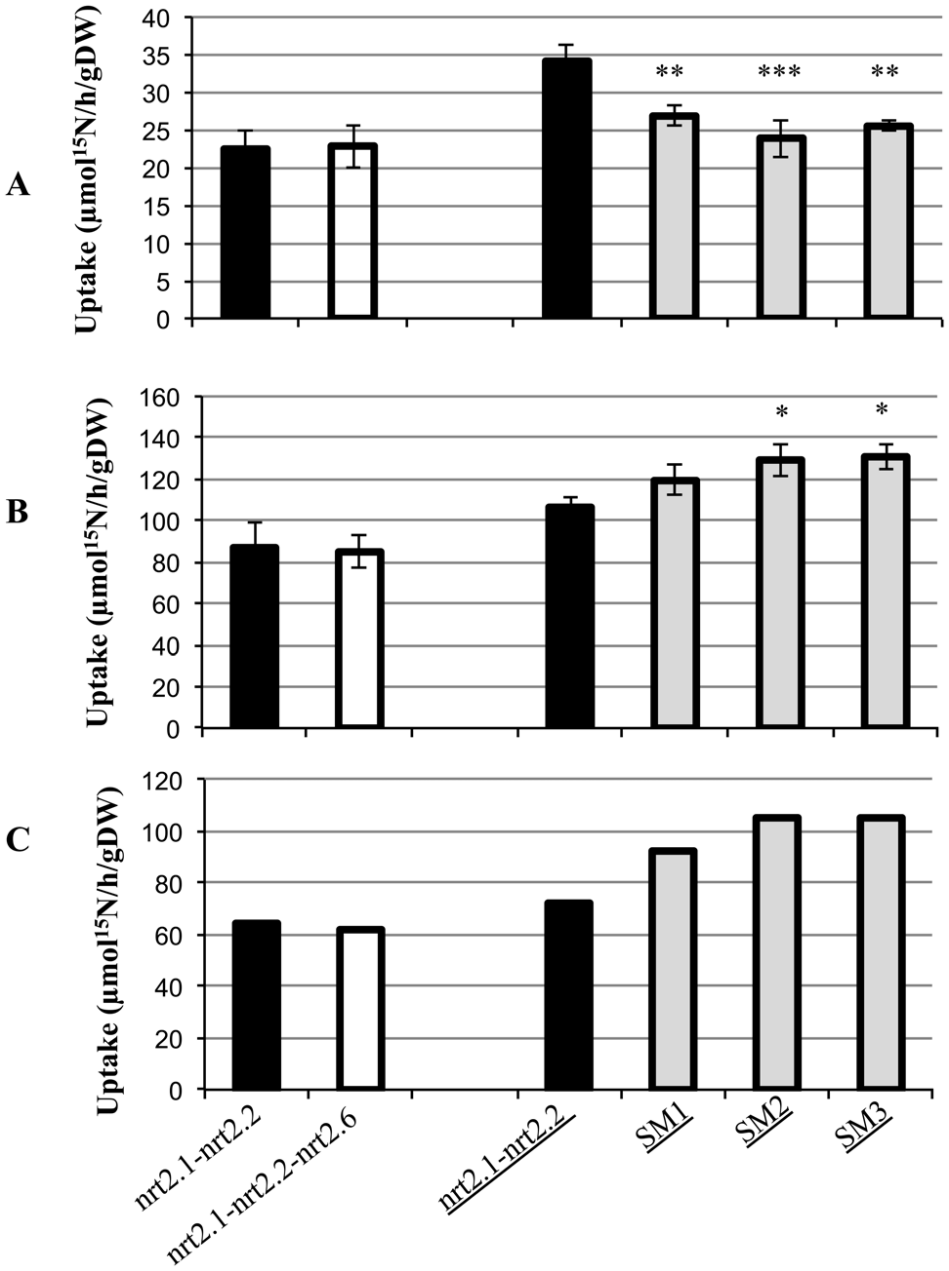
Nitrate uptake in different genotypes. Mutants and supplemented mutants (SM) in Col or Ws (underlined) genetic backgrounds were grown in hydroponic culture on 0.5 mM NH_4_NO_3_ and transferred for 1 additional week to 0.2 mM NO_3_^−^. Root influx was measured at both 0.2 and 6 mM ^15^NO_3_^−^ to provide estimation of HATS (**A**) and HATS + LATS (**B**) activities, respectively. LATS activities (**C**) were calculated as the difference between HATS + LATS and HATS. The asterisks indicate a statistically significant difference between the SM genotypes and their controls (*nrt2.1-nrt2.2* mutants) (Test Fisher *P<0.05, ** p<0.01, ***P<0.001).

The NRT2.1 protein is the main actor of root nitrate uptake mediated by the HATS and could prevent the detection of a smaller contribution. We thus crossed the *nrt2.6-1* with the *nrt2.1–nrt2.2 (Col)* double mutant, isolated in the Columbia genetic background and affected in the *AtNRT2.1* and *AtNRT2.2* genes, and performed comparative analyses of influx capacities. As shown in figure 4, the triple mutant, *nrt2.1-nrt2.2-nrt2.6* had the same HATS and LATS capacities as the *nrt2.1-nrt2.2* double mutant. As the *NRT2.6* is expressed only weakly in roots (Figure 1), we then overexpressed the protein in the *nrt2.1-nrt2.2 (Ws)* double mutant isolated in Ws genetic background [4] with the *NRT2.6* coding sequence under the control of a strong promoter (35S) (Figure 4, A, B and C, underlined genotypes). Following 0.2 mM ^15^NO_3_^−^ supply, the three supplemented lines (SM) showed a significant decrease of HATS-mediated nitrate uptake in roots, about 21% in SM1, 30% in SM2 and 27% in SM3, in comparison to *nrt2.1-nrt2.2*. Conversely, with 6 mM ^15^NO_3_^−^ supply, two of the three supplemented mutants, SM2 and SM3, show a slight significant increase in HATS + LATS activities of 22% in comparison to *nrt2.1-nrt2.2* genotype. When LATS activity was measured as the difference between root ^15^NO_3_^−^ influx measured at 6 mM and 0.2 mM, a slight increase was observed for SM genotypes in comparison to the mutant (Figure 4C). Thus, the *NRT2.6* gene does not seem to be able to complement the nitrate uptake defect of *nrt2.1-nrt2.2* mutant even when it is overexpressed in roots. Moreover, this overexpression led to a slight decrease in HATS capacity.

### The Expression of *NRT2.6* is Induced Upon *E. amylovora* Infection

**Figure 5:**
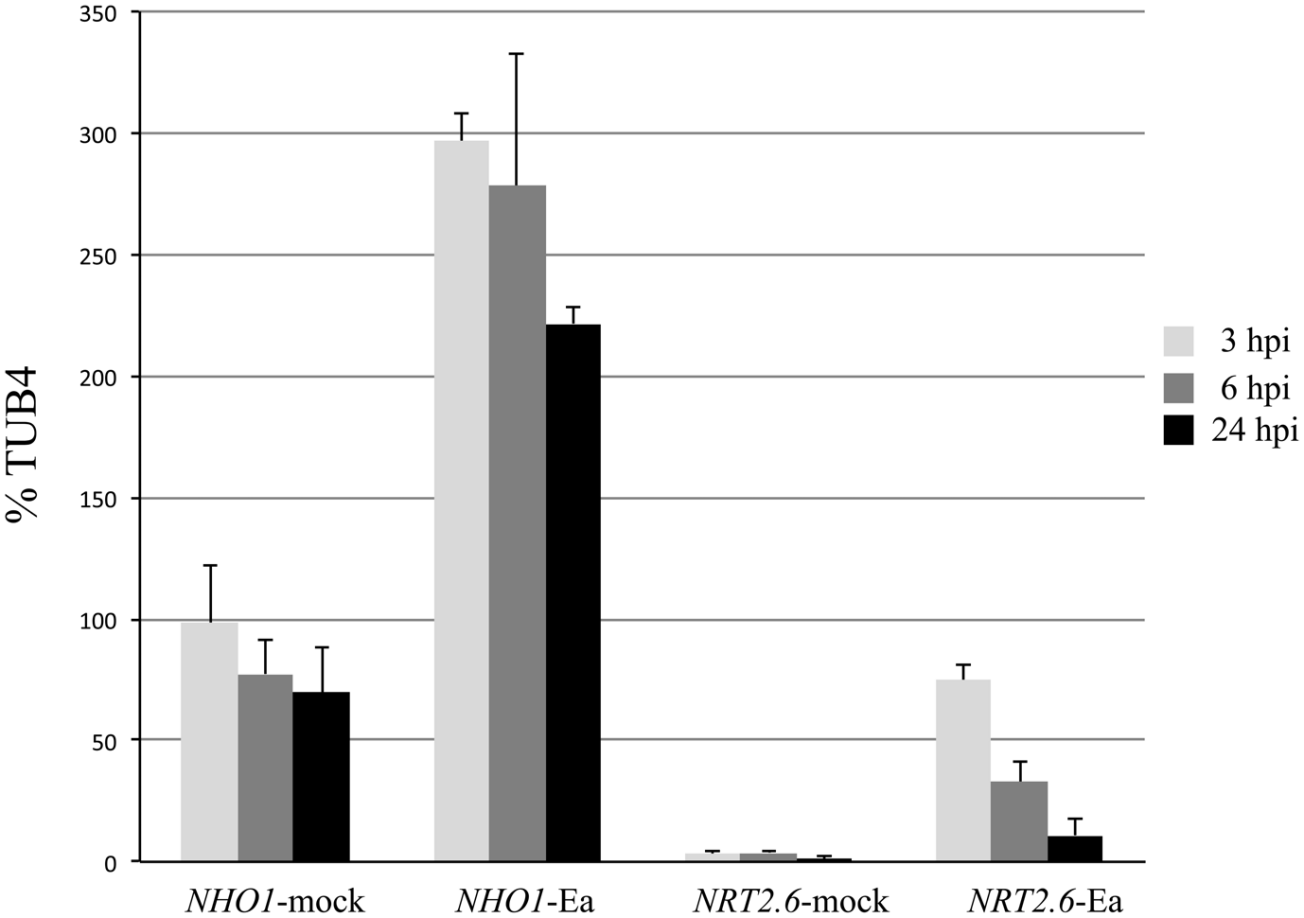
Time course of *NHO1* and *NRT2.6* expression following *E amylovora* inoculation. Plants were grown in culture chamber and leaves were inoculated with wild type *E. amylovora* strain (Ea) or with water (mock) as described in Materials and Methods. RT-qPCR analyses were performed on inoculated leaves 3, 6 or 24 h post inoculation (hpi).

CATMA microarray data from the public resource of Arabidopsis expression database CATdb (urgv.evry.inra.fr/cgi-bin/projects/CATdb/catdb_index.pl) show that the *NRT2.6* gene responds to few stimuli. The strongest induction is found in response to *E. amylovora* infection. *E. amylovora* is a pathogenic bacterium causing fire blight disease on members of the rosaceae family such as apple and pear trees. Several of the host reactions have been also found in Arabidopsis in which *E. amylovora* triggers a type three secretion system (T3SS)–dependent cell death [30]. This interaction leads to leaf necrosis, which is correlated with bacterial growth (M Fagard, unpublished data). To confirm the public CATMA data, we checked the expression of *NRT2.6* following *E. amylovora* inoculation (Figure 5). The *NRT2.6* expression was induced by the bacteria as soon as 3 h post inoculation (hpi), and subsequently decreased. This short-term response can be compared with the expression of an early responsive gene to pathogen attack like *Non-Host 1* [*NHO1*, 31].

### Role of *NRT2.6* in Plant Response to *E. amylovora*

**Figure 6:**
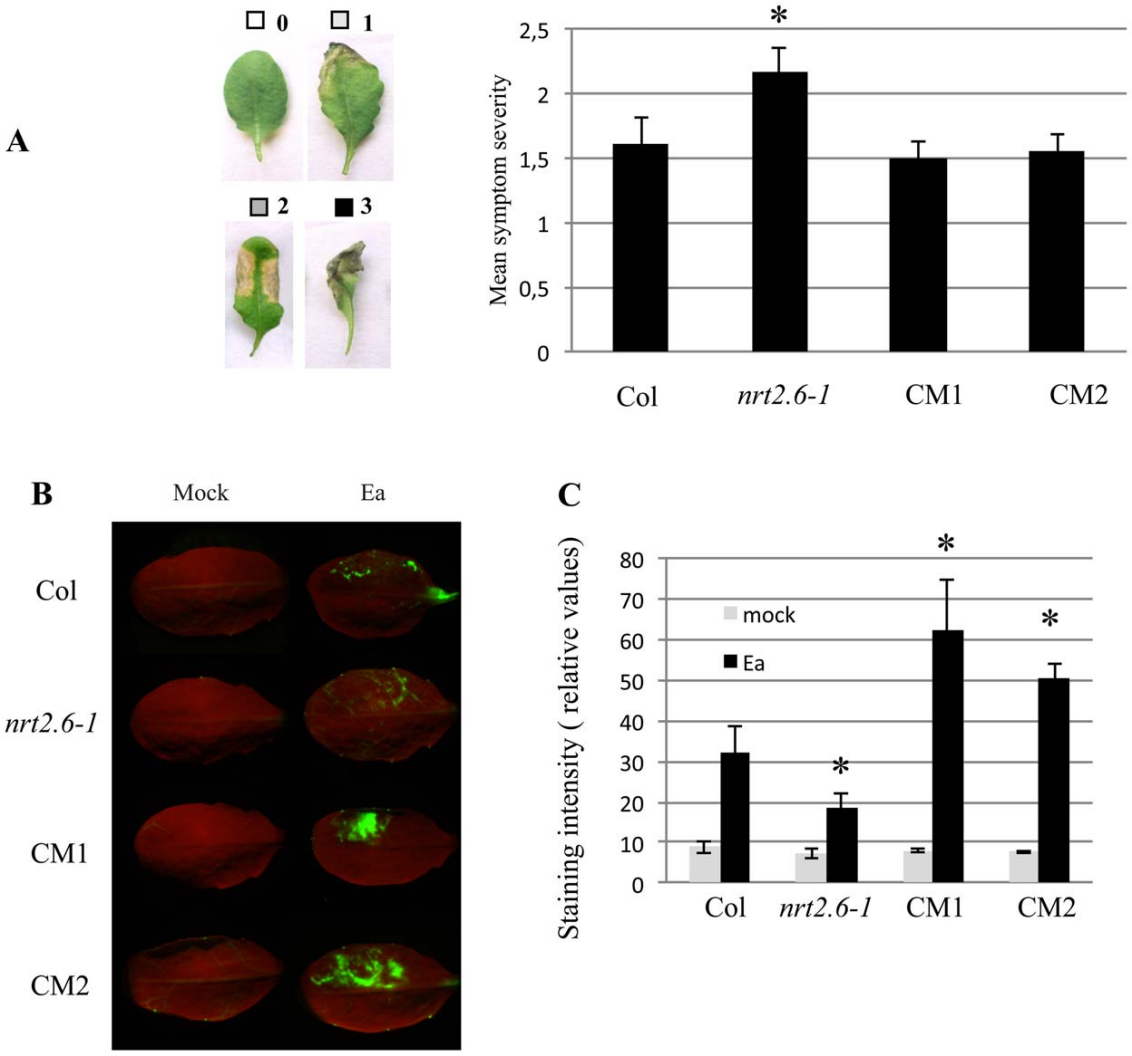
Responses of different genotypes to *E. amylovora* inoculation. Col: Columbia wild-type, *nrt2.6-1*: *NRT2.6* mutant, CM1 and CM2: *nrt2.6-1* mutant complemented by 35S::*NRT2.6* construct. **A**: Mean symptom intensities induced on *A. thaliana* leaves 48 h after *E. amylovora* inoculation. **B**: Accumulation of ROS detected 18 h after *E. amylovora* inoculation by DCFH-DA coloration and microscope imaging. **C**: Quantitative analysis of staining intensities. The asterisk indicates means that are statistically different from wild-type according to Mann and Whitney’s test (*P value <0.05*).

We further investigated the potential role of *NRT2.6* during the plant’s response to *E. amylovora* inoculation using three different *NRT2.6*–related genotypes. As there was only one mutated allele in the Arabidopsis mutant libraries, we complemented the *nrt2.6-1* mutant with the *NRT2.6* coding sequence under the control of a strong promoter (35S). Two complemented mutants, CM1 and CM2, accumulating *NRT2.6* mRNAs 126 and 238 times more than the wild-type respectively (Figure S3), were then analysed. Two days after *E. amylovora* inoculation, *nrt2.6-1* exhibited significantly stronger symptoms than the wild-type (Col). These symptoms were correlated with a higher bacterial multiplication in the mutant than in the wild-type (Figure S4). In contrast to *nrt2.6-1*, the CM lines displayed wild-type levels of necrotic symptoms in response to the pathogen (Figure 6A). Therefore, the altered phenotype of the *nrt2.6-1* mutant in response to *E. amylovora* infection can be attributed to the loss of *NRT2.6* function since its overexpression in the two complemented mutant lines is able to reverse the phenotype.

It is known that in apple leaves, the infection by *E. amylovora* is associated with the activation of the expression of defense genes [32], [33]. The same defense signaling pathways take place after *E. amylovora* inoculation in Arabidopsis [30]. To dissect the response of *nrt2.6-1* mutant and CM lines to *E. amylovora* infection, we chose to study the expression of the following marker genes: *AtrbohD* (Respiratory Burst Oxydase Homologue D), known to be involved in ROS production, *SID2* (Salicylic acid Induction Deficient 2), coding for an isochorismate synthase of the salicylic acid (SA) synthesis pathway, *PR2* (Pathogenesis Related protein 2) also called BGL2 (ß-Glucanase 2) which has been associated with prograµmed cell death (PCD), *PR1* (Pathogenesis Related protein 1), an SA-induced gene and *NHO1* (Non-Host 1). Figure S5 shows that the expression of *NHO1*, *AtrbohD*, *PR2* and *SID2* were found to be similar in all the genotypes, including the wild-type, 24 h post *E. amylovora* inoculation.

As the expression of defense genes did not change after *E. amylovora* inoculation in our genotypes, we measured cellular defense responses like callose deposition (Figure S6), nitric oxide (NO) production and ROS accumulation. We did not detect any significant difference between our genotypes for the first two traits. But, when we analyzed the H_2_O_2_ production at 18 hpi in inoculated leaves by DCFH-DA (2,7-Dichlorodihydrofluorescein diacetate) coloration (Figure 6B), a significant difference appeared between wild-type, mutant and CM lines (Figure 6C). The *nrt2.6-1* mutant accumulated less H_2_O_2_ than Col whereas the H_2_O_2_ accumulation was stronger in the CM lines as compared to wild-type.

Therefore, although the expression of defense responsive genes was not modified in our genotypes following *E. amylovora* inoculation, one of the plant defense responses is modified: the amount of H_2_O_2_ is decreased in the mutant and increased in the complemented mutants.

### Role of *NRT2.6* in Plant Response to Oxidative Stress

**Figure 7:**
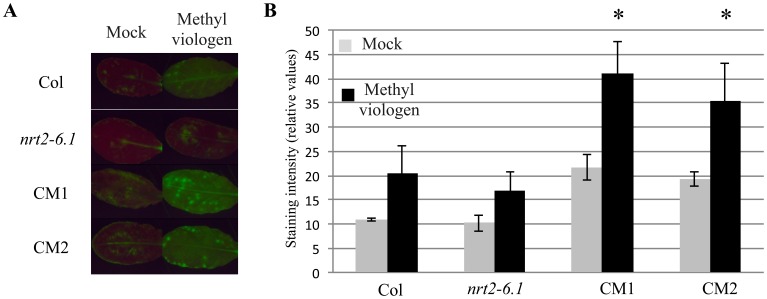
Responses of different genotypes to spray of methyl viologen. Leaves of 5 week-old plants grown in the greenhouse were sprayed with a solution containing 0.1 µM methyl viologen or with water (mock). Col: Columbia wild-type, *nrt2.6-1*: *NRT2.6* mutant, CM1 and CM2: *nrt2.6-1* mutant complemented by 35S::*NRT2.6* construct. **A**: Representative pictures of ROS accumulation detected by DCFH-DA 3 h after treatment. **B**: Quantitative analysis of staining intensities. The asterisks indicate means that are statistically different from the mutant according to Mann and Whitney’s test (*P value <0.05).*

In addition to being produced during plant pathogen interactions, active oxygen species can be produced and accumulate after certain drug treatments. For example, methyl viologen, used as an herbicide, is a redox-active compound that generates superoxide anions in chloroplasts [34]. To test the response of the *NRT2.6* modified genotypes to methyl viologen, we performed leaf inoculation of different drug concentrations (0.05, 0.1 and 0.25 µM) by syringe injections or reagent spraying. We found that H_2_O_2_ was produced as soon as 30 min after inoculation in a methyl viologen dose-dependent manner and that spraying instead of syringe inoculation led to more reproducible results when compared to water-treated plants (data not shown). Eight leaves corresponding to 4 independent plants of each genotype were sprayed with either 0.1 µM of methyl viologen or water and DCFD-DA coloration was performed 3 h later as described in Materials and Methods (Figure 7A). For all four genotypes, no more than one leaf showed faint fluorescence signals after water spraying. In contrast, in response to methyl viologen treatment, 25% and 12.5% of wild-type or mutant leaves showed high accumulation of ROS, respectively, while at least 50% of overexpressor leaves exhibited strong fluorescence after DCFH-DA coloration. These results were quantified by measuring staining intensities (Figure 7B) and statistically significant differences appeared between the two CM complemented lines and the *nrt2.6-1* mutant.

Altogether, our data suggest that the function of NRT2.6 is positively correlated with the accumulation of H_2_O_2_.

## Discussion

Among the seven Arabidopis *NRT2* genes, the roles of *NRT2.1* [5], *NRT2.2* [5], *NRT2.4* [12] and *NRT2.7* [13] are clearly established and we were interested in the role of the other family members, particularly the *NRT2.6*. The closest gene to *NRT2.6* (At3g45060) is *NRT2.3*, which shares 91% of nucleotide identity [28], but nothing is known on its nitrate transport capacity and its potential role *in planta*.

### The NRT2.6 Protein is Unable to Complement a Nitrate Uptake Deficient Mutant

A double mutant with a well-characterized nitrate uptake deficiency mutant is a valuable tool to study the capacity of a protein to participate in the nitrate uptake process. For example, the activities of the very close proteins NRT2.1 and NRT2.2 were deciphered by transformation of the corresponding double mutant by a single gene (*NRT2.1*) [5]. The lack of mutant phenotype of *nrt2.6-1* compared to wild-type for nitrate content and nitrate uptake could be easily explained by the very low expression of the gene in the root cells (Figure 1). However, *in planta* experiments using the *nrt2.1-nrt2.2* double mutant failed to demonstrate a direct role in the nitrate transport process for the NRT2.6 protein. Indeed, over expression of *NRT2.6* in the *nrt2.1-nrt2.2* mutant did not bring any evidence of even partial complementation. Rather it led to a decrease in HATS activity (Figure 5), which could suggest a role in nitrate efflux. Two members of the NRT1 (*NRT1.5* and *NAXT1*) family have been shown to participate to nitrate efflux at the root plasma membrane [35], [36]. The activity of NRT2.1 protein depends on the presence of a NAR2/NRT2.1 two-components complex at the plasma membrane [37] and one could imagine that the nitrate transport activity mediated by the NRT2.6 protein depends on a partner protein that would not be expressed enough in *nrt2.1-nrt2.2* mutant background to ensure an efficient transport capacity. However, *NAR2.1* expression level in the *nrt2.1-nrt2.2* mutant is similar to wild-type level [38]. We also could not exclude that NRT2.6 protein might be involved in the transport of other molecules than nitrate, as it is the case for some NRT1 family members [39].

### *NRT2.6* is Involved in Arabidopsis Response to *E. amylovora*

The recognition of bacterial pathogens by receptors leads to MAP kinase activation, defense gene induction, callose deposition, synthesis of the defense hormone SA and production of ROS [40]. Arabidopsis is naturally resistant to *E. amylovora*: bacterial cells are only able to multiply weakly and transiently in Arabidopsis leaves and do not colonize non-inoculated tissue as they do in host plants [30]. However, *E. amylovora* is able to induce in inoculated leaves necrotic symptoms, which are correlated with bacterial growth [31], [41], this work. Except for MAP kinase activation which was not tested in this plant-pathogen interaction, all the mechanisms identified on host plants have been shown to take place in the Arabidopsis/*E. amylovora* interaction [30]. The *NRT2.6* gene is expressed very early after bacteria inoculation but is not involved in the subsequent cascade of defense gene induction. Callose deposition was also not altered in the *nrt2.6-1* mutant, showing that it is still able to recognize *E. amylovora* and to set up a partial defense response.

ROS are known to play an important role in plant-pathogen interactions during which they are involved in both signaling and direct antimicrobial activities [42]. We used DCFH-DA to detect H_2_O_2_ and we found a negative correlation between ROS accumulation and bacterial multiplication as well as associated necrotic symptoms. Indeed, ROS accumulation was significantly reduced in the mutant, which showed an increased sensitivity to *E. amylovora* infection (stronger symptoms and higher levels of bacteria). The level of plant sensitivity to bacteria can be compensated by the overexpression of *NRT2.6* in the mutant background, demonstrating the role of the gene in the mutant phenotype. Therefore, our data suggest that ROS production detected by DCFH-DA in Arabidopsis could be correlated with defense against *E. amylovora*.

### Is *NRT2.6* Involved Only in H_2_O_2_ Accumulation?

H_2_O_2_ accumulation in *nrt2.6-1* complemented mutant lines occurred in response to ROS generating treatments, whether they are biotic or abiotic such as methyl viologen spraying. Due to its toxic aspect, the steady-state levels of ROS must be tightly regulated in wild-type plants by a fine tuning between ROS-scavenging and ROS-producing proteins. In Arabidopsis, at least 152 genes are involved in this equilibrium [43]. For example, to disrupt the H_2_O_2_ balance, either the expression of genes involved in H_2_O_2_ production is enhanced or the expression of genes involved in H_2_O_2_ scavenging is inhibited. However, our current knowledge does not allow us to favor one of the two possibilities concerning the potential role of *NRT2.6* regarding ROS homeostasis.

One can ask the question of whether or not *NRT2.6* is involved in other ROS species accumulation? In particular, NO acts as endogenous mediator in different biological processes [44]. For example, the accumulation of NO-related species has been shown to occur intra- and extra-cellularly in tobacco cells in response to cryptogein exposure [45]. Although the understanding of the biosynthesis of NO in plants is still incomplete [46], the best characterized pathway of NO production in plants is through the activity of nitrate reductase (NR). Indeed, the NR deficient *nia1 nia2* double mutant shows reduced NO [20]. We measured by DAF-2DA coloration method, which has been developed as a specific indicator for this molecule, the NO produced during *E. amylovora*/Arabidopsis interactions and found no significant difference between our different genotypes (data not shown). On the contrary, a clear difference appeared with the DCFH-DA coloration test, which allows the detection of intracellular H_2_O_2_ but also the detection of peroxynitrite, a toxic derivative of NO, *in vitro* [47]. In response to a transient NO-burst, the cross talk between NO and H_2_O_2_ production may have led to an elevated level of H_2_O_2_ and of peroxynitrite as well [48].

To further investigate the relationship between nitrate transporters and plant response to pathogens, it will be very interesting on one hand to test if other NRT2 members could be also involved in Arabidopsis-*E. amylovora* interaction. On the other hand, we would like now to explore the potential role of NRT2.6 protein in Arabidopsis interaction with other pathogens like *P. syringae* as it has been performed for NRT2.1 [23].

## Materials and Methods

### Plant Material and Growth Conditions

The *nrt2.6-1* mutant (SM_3.35179) was obtained from the NASC center among a mutagenized population (SM lines transposon) of the Col8 Arabidopsis accession [29]. Homozygous mutant plants were identified by PCR using the forward primer SM-upper (5′-TCAAACCACTATCATTCGCTAAACC-3′) and the reverse specific SM lines transposon primer Spm32 (5′-TACGAATAAGAGCGTCCATTTTAGAGTGA-3′). The mutant was backcrossed two times with the wild type.

Plants were grown *in vitro* (16 h light/8 h dark) with a constant temperature of 25°C and a light intensity of 80 µmol m^−2^ s^−1^. Basic medium without nitrogen contained: 0.8 ‰ Bromo-Cresol Purple (BCP), 0.07% 2–(N-Morpholino) ethanesulfonic (MES) acid pH 6, 2.5 mM KH_2_PO_4_, 2 mM MgSO_4_, 5 mM KCl, 2 mM CaCl_2_, 554 µM myo-inositol, 2.1 µM calcium pantothenate, 8.12 M nicotinic acid, 5.9 µM pyridoxine•HCl, 3.32 µM thiamine•HCl, 0.4 µM biotin, 70 µM H_3_BO_3_, 14 µM MnCl_2_, 0.5 µM CuSO_4_, 10 µM NaCl, 1 µM ZnSO_4_, 0.01 µM CoCl_2_, 0.2 µM NaMoO_4_, 5 ‰ Iron ammoniac citrate, 0.7% agarose (Kalys) and 1% sucrose. In the 1 mM NO_3_ medium, KCl and CaCl_2_ were lowered to 4.5 mM and 1.75 mM, respectively, and 0.5 mM KNO_3_ and 0.25 mM Ca(NO_3_)_2_ were added. For the 9 mM and 18 mM NO_3_ media, KCl was replaced by 5 mM and 10 mM KNO_3_, and CaCl_2_ was replaced by 2 mM and 4 mM Ca(NO_3_)_2_, respectively. For the NH_4_^+^ and Gln media, 5 mM (NH_4_)_2_ succinate or 5 mM glutamine, respectively, were added to the basic medium without nitrogen.

In the greenhouse, plants were grown on sand [49] and fed with a solution of 10 mM NO_3_^−^ containing 5 mM KNO_3_, 2.5 mM Ca(NO_3_)_2_, 0.2 mM NaCl, 0.25 mM KH_2_PO_4_, 0.25 mM MgSO_4_, 10 mg/L Fer-EDTA, 243 µM Mo_7_O_24_(NH_4_)_6_, 0.4 µM H_3_BO_3_, 118 µM SO_4_Mn, 10 µM de SO_4_Cu and 34.8 µM SO_4_Zn. Plants were sub-irrigated over 2 h three times per week.

### RNA Extraction and Quantitative PCR (qPCR)

Total RNA was isolated using the Trizol Reagent procedure (Invitrogen). First-strand were synthesised according to Daniel-Vedele and Caboche [50] using M-MLV reverse transcriptase (Gibco-BRL) and oligo (dT) 15 primers. The qPCR was performed on a Mastercycler Realplex instrument (Eppendorf) with the MESA FAST qPCR MasterMix Plus (Eurogentec). Each reaction was performed on a 1/20 dilution of the first cDNA strands in a total reaction of 20 µL. The primers used for qPCR are listed in Table S1.

### Nitrate Measurements

Nitrate was extracted in water from frozen aliquots of plant material. The anion detection was performed with a colorimetric method based on the detection of a chromophore obtained by reduction of nitrate to nitrite by Vanadium (III), adapted from Miranda [51].

### Influx Experiments

Plants were grown for 42 days under hydroponic culture conditions on 0.5 mM NH_4_NO_3_ as previously described [52] (8 h light at 21°C, 150 µmol.m^−2^.s^−1^/16 h dark at 17°C). Nutrient solution was renewed every 2 d and, during the 2 first weeks, used at half-strength. At the age of 5 weeks and 7 d before the experiment, the plants were transferred to basic medium in which N was supplied as 0.2 mM NO_3_^−^, and the solution was changed daily. After 42 days, the plants were transferred first to 0.1 mM CaSO_4_ for 1 min, then to basic nutrient solution containing 0.2 mM ^15^NO_3_^−^ (atom% ^15^N:99%) or 6 mM ^15^NO_3_^−^ for 5 min and finally to 0.1 mM CaSO_4_ for 1 min. After homogenization, an aliquot of the frozen powder was dried overnight at 80°C and analyzed using the ANCA-MS system (PDZ Europa). Influx of ^15^NO_3_^−^ was calculated from the total N and ^15^N content of the roots.

### GUS Construction and Staining

Binary vectors containing *uidA* fusions with the *NRT2.6* promoter were obtained using Gateway technology [53]. A genomic Arabidopsis *NRT2.6* region, starting from position - 2000 bp upstream of the translation initiation site and terminating before the ATG codon, was amplified from Ws accession by PCR with primers NRT2.6 PGW5′ (5′-AAAAAAGCAGGCTAAAGACCATCCCGATGAAAG-3′) and NRT2.6 PGW3′ (5′-AAGAAAGCTGGGTTGTAAGTTGGAGAAGATGAG-3′). Amplification was performed using the Expand high-fidelity PCR system (Roche), and the amplified fragment was cloned in front of the GUS coding sequence in the pBI101 derived gateway vector [54]. The binary plasmids were transferred to *Agrobacterium tumefaciens* strain C58C1 (pMP90) by triparental mating. Wild type Arabidopsis plants, were transformed according to the *in planta* method using the surfactant Silwet L-77 [55]. Transgenic plants were selected on Estelle and Sommerville media [56] containing 50 mg.L^−1^ of kanamycin.

The transgenic plants carrying the *ProNRT2.6*::*uidA* construct were grown on horizontal plates [56] at 25°C under long-day conditions or in the greenhouse. The plants were observed under a light microscope (Axioplan 2; Zeiss) after GUS staining [57]. For histological analysis, samples were embedded in resin as already described [58] and blocks were sectioned at 4 µm thickness using a Leica RM 2165 microtome.

### Generation of Complemented Lines

First primers AttB1-NRT2.6start (5′-GGAGATAGAACCATGGCTCACAACCATTCTAATG) and AttB2-NRT2.6end-stop (5′-TCCACCTCCGGATCAGACATGAGCCGGAGATCC-3′) were used to amplify a complete *NRT2.6* cDNA from roots of 33 day-old Columbia plants. PCR products were obtained with the iProof High Fidelity PCR kit (Bio-Rad) and amplified with the universal U3endstop (5′-AGATTGGGGACCACTTTGTACAAGAAAGCTGGGTCTCCACCTCCGGATC-3′) and U5 primers (5′-GGGGACAAGTTTGTACAAAAAAGCAGGCTTCGAAGGAGATAGAACCATG-3′) to create the recombinant site AttB. The product of recombination reactions (BP reactions) was used to transform competent *Escherichia coli*, strainTOP10 (Invitrogen), by heat shock. LR clonase reactions to transfer T-DNA fragments from the entry clone to the destination binary vector pMDC32 [53] were performed. The vector pMDC32/*NRT2.6* was generated and the binary vector, containing the *Pro35S*::*NRT2.6* construct was sequenced before transformation of *A. tumefaciens*. The *nrt2.6-1* and *nrt2.1-nrt2.2* double mutants were transformed with the pMDC32/*NRT2.6* constructs by the *in planta* method using the surfactant Silwet L-77 [55] and transformants were selected on 20 mg.L^−1^ of hygromycin B.

### *E. amylovora* Inoculations, Methyl-viologen Treatments and ROS Detection

Arabidopsis plants were grown in the greenhouse as described [30] in an 8 h light/16 h dark cycle, at 19°C, with 70% relative humidity, using 10 mM NO_3_^−^ as nitrogen source during 5 weeks. Inoculations were performed using a blunt syringe with the bacterial wild-type strain *E. amylovora* CFBP1430 [59] and the inoculum density was adjusted at 0.1 O.D. in water (10^7^ c.f.u.mL^−1^). Symptom severity was scored according to a visual scale from 0 (no apparent necrosis) to 3 (necrosis of the whole leaf) as described in Degrave et al [30]. Bacterial growth was analyzed 24 h after inoculation as described by Degrave et al [30].

For methyl viologen treatments, two rosette leaves by plant were inoculated by one spray with a water solution containing different concentrations of methyl viologen (Acros-Organics, from 0.05 µM to 0.25 µM). Water atomization was used as a control and, 30 min after treatment, leaves were subjected to DCFH-DA coloration.

ROS detection method was adapted from Zhang et al [60]. At 18 h following half-leaf infiltration with E. amylovora or water, leaves were immersed in a 300 µM DCFH-DA (2,7-Dichlorodihydrofluorescein-diacetate) solution and vacuum-infiltrated. Whole leaf images were taken using an Olympus SZX12 binocular magnifier. Green fluorescence was detected with an HQ510 1p emission filter. Experiments were repeated twice and quantitative measurements were done by measuring mean gray levels of the green channel of each image by using ImageJ v1.46f (Rasband, W.S., ImageJ, U. S. National Institutes of Health, Bethesda, Maryland, USA, http://imagej.nih.gov/ij/, 1997–2011).

### Analysis of Callose Accumulation

For callose detection, the leaves were inoculated as described above and collected 8 hpi. Callose deposits were detected using aniline blue as described in [30]. Experiments were repeated twice with similar results. Representative pictures are shown. The number of callose deposits per picture was determined using ImageJ (National Institute of Health, Bethesda, MD, U.S.A.) and compared using Mann and Whitney’s test (∝* = 0.05*). We analyzed 25 to 30 pictures corresponding to more than five independent leaves for each treatment.

## Supporting Information

Figure S1 **Role of** ***NRT2.6*** **in the tapetum.** **A:** Transmission of T-DNA to the progeny. Mother plants grown in the greenhouse were fed with 10 mM nitrate until bolting and then 0.2, 2, 10 and 50 mM nitrate until seed maturation. Seeds were sown on agar medium containing basic medium with 9 mM NO_3_^−^ as sole nitrogen source. T-DNA or native gene was detected by PCR analyses. **B**: Pollen viability measured by Alexander test. Alexander test was performed on opened flowers from wild type and mutant [61].
(TIF)

Figure S2 **Structure of the transposon insertion in the** ***nrt2.6-1*** **mutant.**
(TIFF)

Figure S3 **Levels of** ***NRT2.6*** **expression in two complemented lines.** Plants were grown under standard conditions in the greenhouse and transgene *NRT2.6* expression was measured by RT-qPCR as described in Materials and Methods. An arbitrary value of 1 was given to *NRT2.6* expression in Col.
(TIF)

Figure S4 **The** ***nrt2.6-1*** **mutant supports higher bacterial multiplication of** ***E. amylovora cells*** **than wild-type plants.** Bacterial count of *E. amylovora* in wild-type (Col) and mutant (*nrt2.6-1*) plants. The number of CFU present in leaf extracts was counted 24 h post inoculation. The asterisk indicates that the means are statistically different according to Mann and Whitney’s test (*P value <0.05).*
(TIF)

Figure S5 **Defense gene expression in response to infection by** ***E. amylovora*** **.** Expression of marker genes was measured 24 h after *E. amylovora* inoculation. A 100% arbitrary value was affected to expression levels in Col.
(TIF)

Figure S6 **Callose accumulation in response to** ***E. amylovora*** **is not affected in the** ***nrt2.6-1*** **mutant.** Analysis of callose deposits in *E. amylovora*-inoculated wild-type (Col) and mutant (*nrt2.6-1*) plants. Leaves were collected 8 hpi and stained with aniline blue as described previously [31]. No significant difference in callose deposition could be observed between wild-type and mutant plants. **A**: Representative images are shown for each treatment. **B**: Experiments were repeated twice with similar results. The asterisks indicate that the means are statistically different between mock and Ea treatments according to Mann and Whitney’s test (*P value <0.05).* No statistical differences were found between wild-type and *nrt2.6-1* mutant.
(TIF)

Table S1 **Sequences of oligonucleotides used in RT-PCR reactions.** This table summarizes the sequences of oligonucleotides used in this study. For each targeted gene, the sequences of forward (F) primer and reverse (R) primer are given.
(TIF)

## References

[1] Tsay YF, Chiu CC, Tsai CB, Ho CH, Hsu PK (2007) Nitrate transporters and peptide transporters. FEBS Lett 58: 2290–2300. 10.1016/j.febslet.2007.04.047 17481610

[2] Dechorgnat J, Nguyen CH, Armengaud P, Jossier M, Diatloff E, et al (2011) From the soils to the seeds: the long journey of nitrate in plants. J Exp Bot 62: 1349–1359. 21193579 10.1093/jxb/erq409

[3] Glass ADM, Sidiqui MY (1995) Nitrogen absorption by plant roots. In *Nitrogen nutrition in higher plants* - Srivastava HS, Singh RP, eds. Associated Publishing Co, New Delhi India, 21–56.

[4] Filleur S, Dorbe MF, Cerezo M, Orsel M, Granier F, et al (2001) An Arabidopsis T-DNA mutant affected in *NRT2* genes is impaired in nitrate uptake. FEBS Lett 489: 220–224. 11165253 10.1016/s0014-5793(01)02096-8

[5] Li W, Wang Y, Okamoto M, Crawford NM, Siddiqi MY, et al (2007) Dissection of the *AtNRT2.1*:*AtNRT2.2* inducible high-affinity nitrate transporter gene cluster. Plant Physiol 143: 425–433. 17085507 10.1104/pp.106.091223 PMC1761961

[6] Filleur S, Daniel-Vedele F (1999) Expression analysis of a high-affinity nitrate transporter isolated from *Arabidopsis thaliana* by differential display Planta. 207: 461–469. 10.1007/s004250050505 9951738

[7] Lejay L, Wirth J, Pervent M, Cross JM, Tillard P, et al (2008) Oxidative pentose phosphate pathway-dependent sugar sensing as a mechanism for regulation of root ion transporters by photosynthesis. Plant Physiol 146: 2036–2053. 18305209 10.1104/pp.107.114710 PMC2287369

[8] Vidmar JJ, Zhuo D, Siddiqi MY, Schjoerring JK, Touraine B, et al (2000) Regulation of high-affinity nitrate transporter genes and high-affinity nitrate influx by nitrogen pools in roots of barley. Plant Physiol 123: 307–318. 10806247 10.1104/pp.123.1.307 PMC59004

[9] Nazoa P, Videmar J, Tranbarger TJ, Mouline K, Damiani I, et al (2003) Regulation of the nitrate transporter gene *AtNRT2.1* in *Arabidopsis thaliana*: responses to nitrate, amino acids and developmental stage. Plant Mol Biol 52: 689–703. 12956537 10.1023/a:1024899808018

[10] Chopin F, Wirth J, Dorbe MF, Lejay L, Krapp A, et al (2007) The Arabidopsis nitrate transporter AtNRT2.1 is targeted to the root plasma membrane. Plant Physiol Biochem 45: 630–63516. 17583518 10.1016/j.plaphy.2007.04.007

[11] Wirth J, Chopin F, Santoni V, Viennois G, Tillard P, et al (2007) Regulation of root nitrate uptake at the NRT2.1 protein level in *Arabidopsis thaliana* . J Biol Chem 282 23541–23552. 17573350 10.1074/jbc.M700901200

[12] Kiba T, Feria-Bourrelier AB, Lafouge F, Lezhneva L, Boutet-Mercey S, et al (2012) The Arabidopsis nitrate transporter NRT2.4 plays a double role in roots and shoots of nitrogen-starved plants. Plant Cell 24: 245–258. 22227893 10.1105/tpc.111.092221 PMC3289576

[13] Chopin F, Orsel M, Dorbe MF, Chardon F, Truong HN, et al (2007) The *Arabidopsis* ATNRT2.7 nitrate transporter controls nitrate content in seeds. Plant Cell 19: 1590–1602. 17540716 10.1105/tpc.107.050542 PMC1913726

[14] Okamoto M, Vidmar JJ, Glass ADM (2003) Regulation of *NRT1* and *NRT2* gene families of *Arabidopsis thaliana*: Responses to nitrate provision. Plant Cell Physiol 44: 304–317. 12668777 10.1093/pcp/pcg036

[15] Fan SC, Lin CS, Hsu PK, Lin SH, Tsay YF (2009) The Arabidopsis nitrate transporter NRT1.7, expressed in phloem, is responsible for source-to-sink remobilization of nitrate. Plant Cell 21: 2750–2761. 19734434 10.1105/tpc.109.067603 PMC2768937

[16] Liu G, Ji Y, Bhuiyan NH, Pilot G, Selvaraj G, et al (2010) Amino acid homeostasis modulates salicylic acid-associated redox status and defense responses in Arabidopsis. Plant Cell 22: 3845–3863. 21097712 10.1105/tpc.110.079392 PMC3015111

[17] Hubert D, Watsonn R (1974) Nitrogen form and plant disease. Annu Rev Phytopathology 12: 139–155. 10.1146/annurev.py.12.090174.001035 23249125

[18] Snoeijers SS, Pérez-Garcia A, Joosten MHAJ, De Witt PJGM (2000) The effect of nitrogen on disease development and gene expression in bacterial and fungal plant pathogens. European J of Plant Pathology 106: 493–506.

[19] Jones DG, Dangl JL (2006) The plant immune system. Nature 444: 323–329.23. 17108957 10.1038/nature05286

[20] Modolo LV, Augusto O, Almeida IMG, Pinto-Maglio CAF, Oliviera HC, et al (2006) Decreased arginine and nitrite levels in nitrate-reductase deficient *Arabidopsis thaliana* plants impair nitrite oxyde synthesis and the hypersensitive response to *Pseudomonas syringae* . Plant Sci 171: 34–40.

[21] Rockel PF, Strube F, Rockel A, Wildt J, Kaiser WM (2002) Regulation of nitric oxyde (NO) production by plant nitrate reductase in vivo and in vitro. J Exp Bot 53: 1–8. 11741046

[22] Oliviera HC, Justino GC, Sodek L, Salgado I (2009) Amino acid recovery does not prevent susceptibility to *Pseudomonas syringae* in nitrate-reductase double-deficient *Arabidopsis thaliana* plants. Plant Sci 176: 105–111.

[23] Camanes G, Pastor V, Cerezo M, Garcia-Andrade J, Vicedo B, et al (2011) A deletion in *NRT2.1* attenuates *Pseudomonas syringae*-induced hormonal perturbation, resulting in primed plant defenses. Plant Physiol 158: 1054–1066. 22158760 10.1104/pp.111.184424 PMC3271742

[24] Deeken R, Engelmann JC, Efetova M, Czirjak T, Müller T, et al (2006) An integrated view of gene expression and solute profiles of arabidopsis tumors : a genome-wide approach. Plant Cell 18: 3617–3634. 17172353 10.1105/tpc.106.044743 PMC1785400

[25] Mantelin S, Desbrosses G, Larcher M, Tranbarger T, Cleyet-Marel JC, et al (2006) Nitrate-dependent control of root architecture and N nutrition are altered by a plant growth-promoting *Phyllobacterium* sp. Planta 223: 591–603. 16160849 10.1007/s00425-005-0106-y

[26] Krapp A, Fraisier V, Scheible WR, Quesada A, Gojon A, et al (1998) Expression studies of *Nrt2:1Np*, a putative high-affinity nitrate transporter: evidence for its role in nitrate uptake. Plant J 14: 723–731.

[27] Goldberg RB, Beals TP, Sanders PM (1993) Anther development: basic principles and practical applications. Plant Cell 5: 1217–1229. 8281038 10.1105/tpc.5.10.1217 PMC160355

[28] Orsel M, Krapp A, Daniel-Vedele F (2002) Analysis of the NRT2 nitrate transporter family in Arabidopsis. Structure and gene expression. Plant Physiol 129: 886–896. 12068127 10.1104/pp.005280 PMC161709

[29] Tissier AF, Marillonnet S, Klimyuk V, Patel K, Torres MA, et al (1999) Multiple independent defective Suppressor-mutator transposable insertions in Arabidopsis: a tool for functional genomics. Plant Cell 11: 1841–1852. 10521516 10.1105/tpc.11.10.1841 PMC144107

[30] Degrave A, Fagard M, Perino C, Brisset MN, Gaubert S, et al (2008) *Erwinia amylovora* type-three-secreted proteins trigger cell death and defense responses in *Arabidopsis thaliana* Mol Plant-Microbe Interact. 21: 1076–1086. 10.1094/MPMI-21-8-1076 18616404

[31] Lu M, Tang X, Zhou JM (2001) Arabidopsis *NHO1* is required for general resistance against Pseudomonas Bacteria. Plant Cell 13: 437–477. 11226196 10.1105/tpc.13.2.437 PMC102253

[32] Venisse JS, Malnoy M, Faize M, Paulin JP, Brisset MN (2002) Modulation of defense response of *Malus spp* during compatible and incompatible interactions with *Erwinia amylovora* . Mol Plant-Microbe Interact 15: 1204–1212. 12481992 10.1094/MPMI.2002.15.12.1204

[33] Bonasera JM, Kim JF, Beer SV (2006) PR genes of apple: identification and expression in response to elicitors and inoculation with *Erwinia amylovora* . BMC Plant Biol 6: 23. 17029637 10.1186/1471-2229-6-23 PMC1613244

[34] Dodge A (1994) Herbicide action and effects on detoxification processes. *In* CH Foyer, PM Mullineaux, eds, Causes of Photooxidative Stress and Amelioration of Defense Systems in Plants. CRC Press, Boca Raton, FL, 219–23.

[35] Lin SH, Kuo HF, Canivenc G, Lin CS, Lepetit M, et al (2008) Mutation of the Arabidopsis NRT1.5 Nitrate Transporter Causes Defective Root-to-Shoot Nitrate Transport. Plant Cell 20: 2514–2528. 18780802 10.1105/tpc.108.060244 PMC2570733

[36] Segonzac C, Boyer JC, Ipotesi E, Szponarski W, Tillard P, et al (2007) Nitrate efflux at the root plasma membrane: identification of an Arabidopsis excretion transporter. Plant Cell 19: 3760–3777. 17993627 10.1105/tpc.106.048173 PMC2174868

[37] Yong Z, Kotur Z, Glass ADJ (2010) Characterization of an intact two-component high-affinity nitrate transporter from Arabidopsis roots. Plant J 63: 739–748. 20561257 10.1111/j.1365-313X.2010.04278.x

[38] Orsel M, Chopin F, Leleu O, Smith SJ, Krapp A, et al (2006) Characterization of a two component high-affinity nitrate uptake system in Arabidopsis. Physiology and protein-protein interactions. Plant Physiol 42 1304–1317. 10.1104/pp.106.085209 PMC1630756 17012411

[39] Song W, Steiner HY, Zhang L, Naider F, Stacey G, et al (1996) Cloning of a second *Arabidopsis* peptide transport gene. Plant Physiol 110: 171–178. 8587981 10.1104/pp.110.1.171 PMC157706

[40] Schewessinger B, Zipfel C (2008) News from the frontline: recent insights into PAMP-triggered immunity in plants. Curr Opin Plant Biol 11: 389–395. 18602859 10.1016/j.pbi.2008.06.001

[41] Moreau M, Degrave A, Vedel R, Bitton F, Patrit O, et al (2012) EDS1 contributes to non-host resistance of *A. thaliana* against *E. amylovora*. Mol Plant-Microbe Interact. 25: 421–430. 10.1094/MPMI-05-11-0111 22316300

[42] Torres MA (2010) ROS in biotic interactions Physiol Plant. 138: 414–429. 10.1111/j.1399-3054.2009.01326.x 20002601

[43] Mittler R, Vanderauwera S, Gollery M, Van Breusegem F (2004) Reactive oxygen network of plants. Trens Plant Sci 9: 490–498. 10.1016/j.tplants.2004.08.009 15465684

[44] Besson-Bard A, Pugin A, Wendehenne D (2008) New insights into nitric oxide signalling in plants. Annu Rev Plant Biol 59: 21–39. 18031216 10.1146/annurev.arplant.59.032607.092830

[45] Besson-Bard A, Griveau S, Bedioui F, Wendehenne D (2008) Real-time electrochemical detection of extracellular nitric oxide in tobacco cells exposed to cryptogein, an elicitor of defense responses. J Exp Bot 59: 3407–3414. 18653691 10.1093/jxb/ern189 PMC2529233

[46] Gupta KJ, Fernie AR, Kaiser WM, van Dongen JT (2011) On the origins of nitric oxide. Trends in Plant Science 16: 160–168. 21185769 10.1016/j.tplants.2010.11.007

[47] Ischiropoulos H, Gow A, Thom SR, Kooy NW, Royall JA, et al (1999) Detection of reactive oxygen species using 2,7-dichlorohydrofluorescein and dihydrorhodamine 123. Meths in Enz 301: 367–373. 10.1016/s0076-6879(99)01100-3 9919585

[48] Zeier J, Delledone M, Mishina T, Severi E, Sonoda M, et al (2004) Genetic elucidation of nitric oxide signaling in incompatible plant-pathogen interactions. Plant Physiol 136: 2875–2886. 15347797 10.1104/pp.104.042499 PMC523349

[49] Chardon F, Barthélémy J, Daniel-Vedele F, Masclaux-Daubresse C (2010) Natural variation of nitrate uptake and nitrogen use efficicnecy in *Arabidopsis thaliana* cultivated with limiting and ample nitrogen supply. J Exp Bot 61: 2293–2302. 20237091 10.1093/jxb/erq059

[50] Daniel-Vedele F, Caboche M (1993) A Tobacco cDNA Clone Encoding a GATA-1 Zinc Finger Protein Homologous to Regulators of Nitrogen Metabolism in Fungi. Mol Gen Genet 240: 365–373. 8413186 10.1007/BF00280388

[51] Miranda KM, Espey MG, Wink DA (2001) A rapid, simple spectrophotometric method for simultaneous detection of nitrate and nitrite. Nitric oxide: Biology and chemistry 5: 62–71. 11178938 10.1006/niox.2000.0319

[52] Merigout P, Lelandais M, Bitton F, Renou JP, Briand X, et al (2008) Physiological and transcriptomic aspects of urea uptake and assimilation in Arabidopsis plants. Plant Physiol 147: 1125–1138. 10.1104/pp.108.119339 PMC2442537 18508958

[53] Curtis MD, Grossniklaus U (2003) A gateway cloning vector set for high throughput functional analysis of genes in planta. Plant Physiol 133: 462–469. 14555774 10.1104/pp.103.027979 PMC523872

[54] Divol F, Vilaine F, Thibivillier S, Kusiak C, Sauge MH, et al (2006) Involvement of the xyloglucan endotransglycosylase/hydrolases encoded by celery XHT1 and Arabidopsis XHT33 in the phloem response to aphids. Plant Cell Env 30: 187–201. 10.1111/j.1365-3040.2006.01618.x 17238910

[55] Clough SJ, Bent AF (1998) Floral dip: a simplified method for *Agrobacterium*-mediated transformation of *Arabidopsis thaliana*. Plant J. 16: 735–743. 10.1046/j.1365-313x.1998.00343.x 10069079

[56] Estelle MA, Sommerville C (1987) Auxin-resistant mutants of *Arabidopsis thaliana* with an altered morphology. Mol. Gen. Genet. 206: 200–206.

[57] Jefferson RA (1987) Assaying Chimeric Gene in Plants: the Gus Gene Fusion System. Plant Mol Biol Rep 5: 387–405.

[58] Jouannic S, Lartaud M, Hervé J, Collin M, Orieux Y, et al (2011) The shoot apical meristem of oil palm (*Elaeis guineensis*. Arecaceae): developmental progression and dynamics. Annals of Bot. 1–11 (doi: 10.1093/aob/mcr019).. 10.1093/aob/mcr019 PMC3219488 21303783

[59] Paulin JP, Samson R (1973) Le feu bactérien en France II Caractères des souches d’*Erwinia amylovora* (Burril) Winslow et al 1920 isolées du foyer franco-belge. Annu Rev Phytopathol 5: 389–397.

[60] Zhang C, Gutsche A, Shapiro AD (2004) Feedback control of the Arabidopsis hypersensitive response. Mol Plant-Microbe Interact 17: 357–365. 15077668 10.1094/MPMI.2004.17.4.357

[61] Alexander MP (1969) Differential staining of aborted and nonaborted pollen. Stain Technol 44: 117–122. 4181665 10.3109/10520296909063335

