# Supplementary figures and images for: Characterization of the Nrt2.6 Gene in Arabidopsis thaliana: A Link with Plant Response to Biotic and Abiotic Stress
Source: PLoS One. 2012 Aug 7;7(8):e42491. doi: 10.1371/journal.pone.0042491 (PMC3413667; doi:10.1371/journal.pone.0042491)

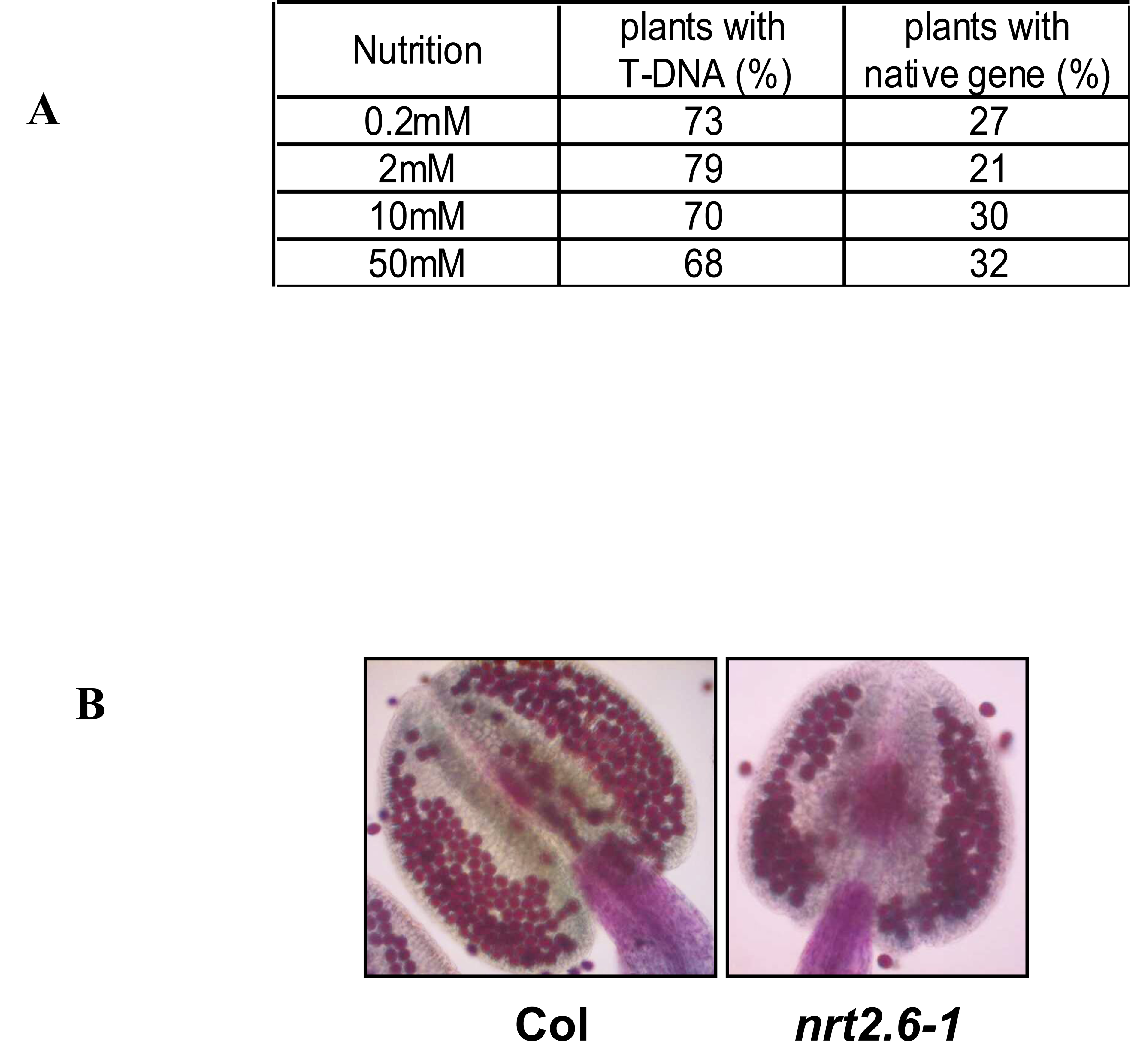

Supplement: Figure S1 — Role of NRT2.6 in the tapetum. A: Transmission of T-DNA to the progeny. Mother plants grown in the greenhouse were fed with 10 mM nitrate until bolting and then 0.2, 2, 10 and 50 mM nitrate until seed maturation. Seeds were sown on agar medium containing basic medium with 9 mM NO3 − as sole nitrogen source. T-DNA or native gene was detected by PCR analyses. B: Pollen viability measured by Alexander test. Alexander test was performed on opened flowers from wild type and mutant [61]. (TIF) [file pone.0042491.s001.tif]

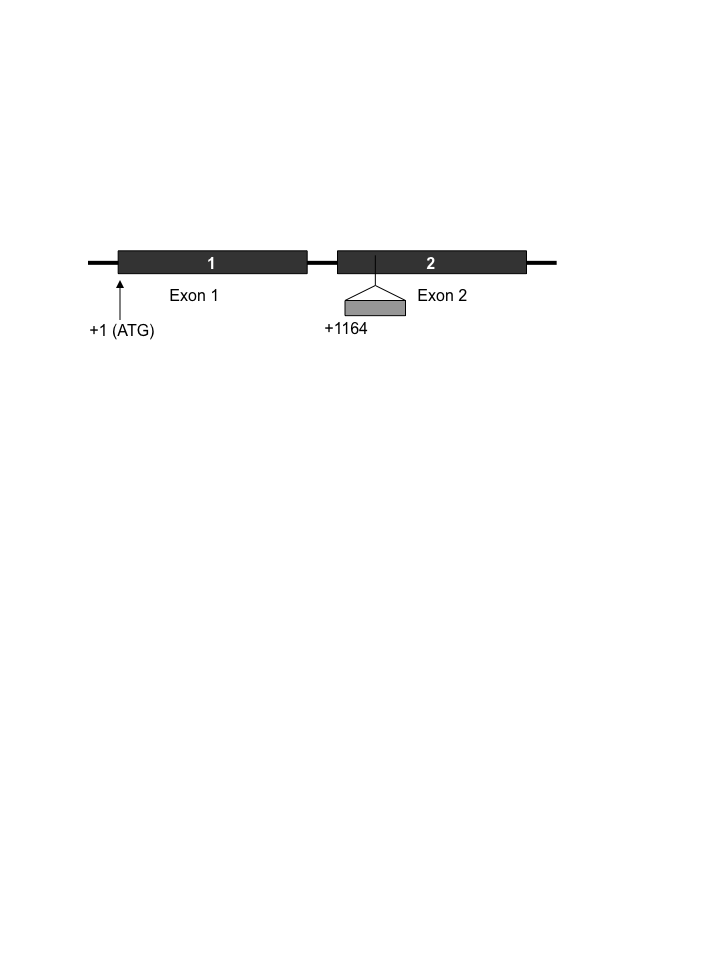

Supplement: Figure S2 — Structure of the transposon insertion in the nrt2.6-1 mutant. (TIFF) [file pone.0042491.s002.tif]

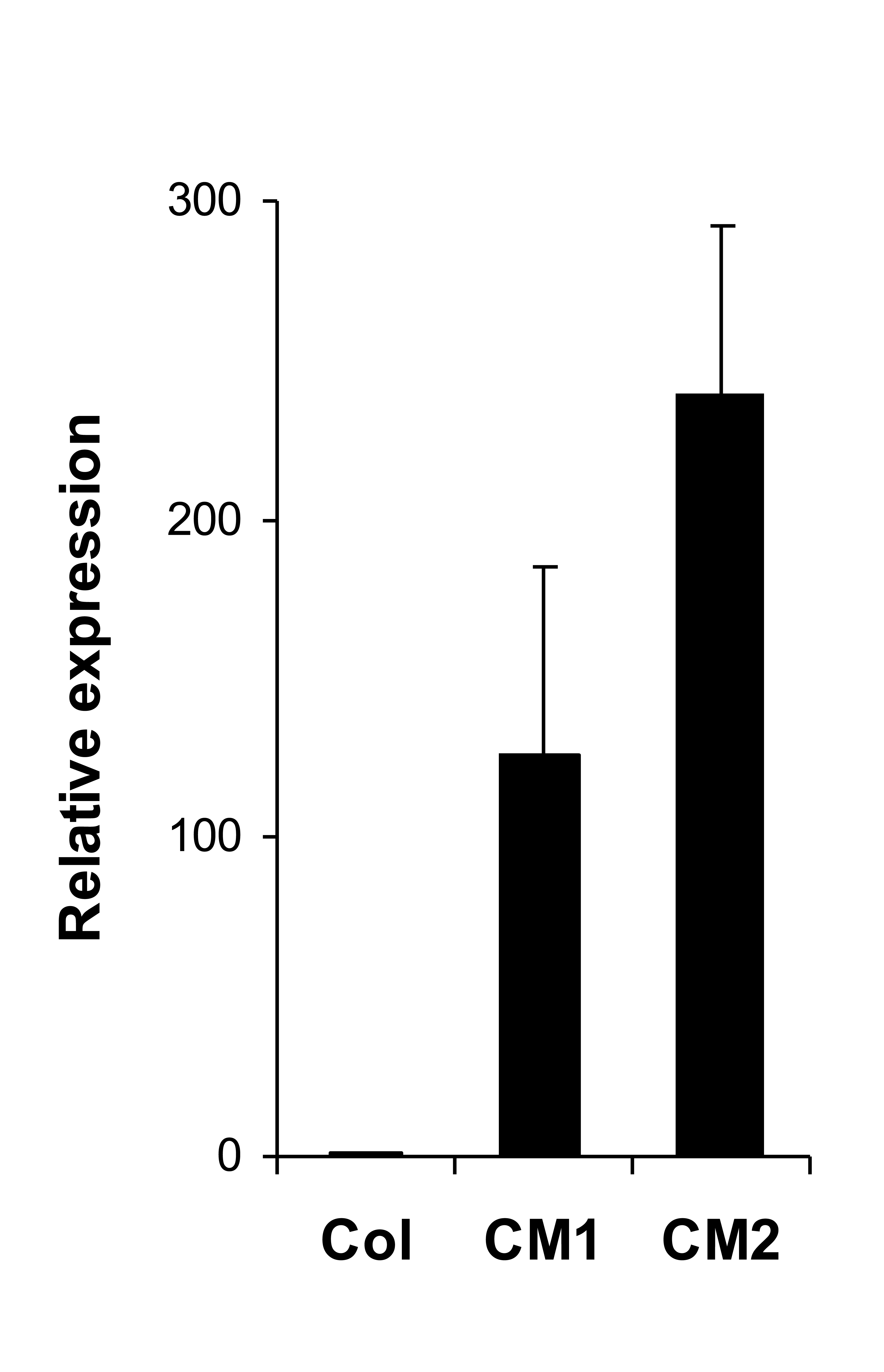

Supplement: Figure S3 — Levels of NRT2.6 expression in two complemented lines. Plants were grown under standard conditions in the greenhouse and transgene NRT2.6 expression was measured by RT-qPCR as described in Materials and Methods. An arbitrary value of 1 was given to NRT2.6 expression in Col. (TIF) [file pone.0042491.s003.tif]

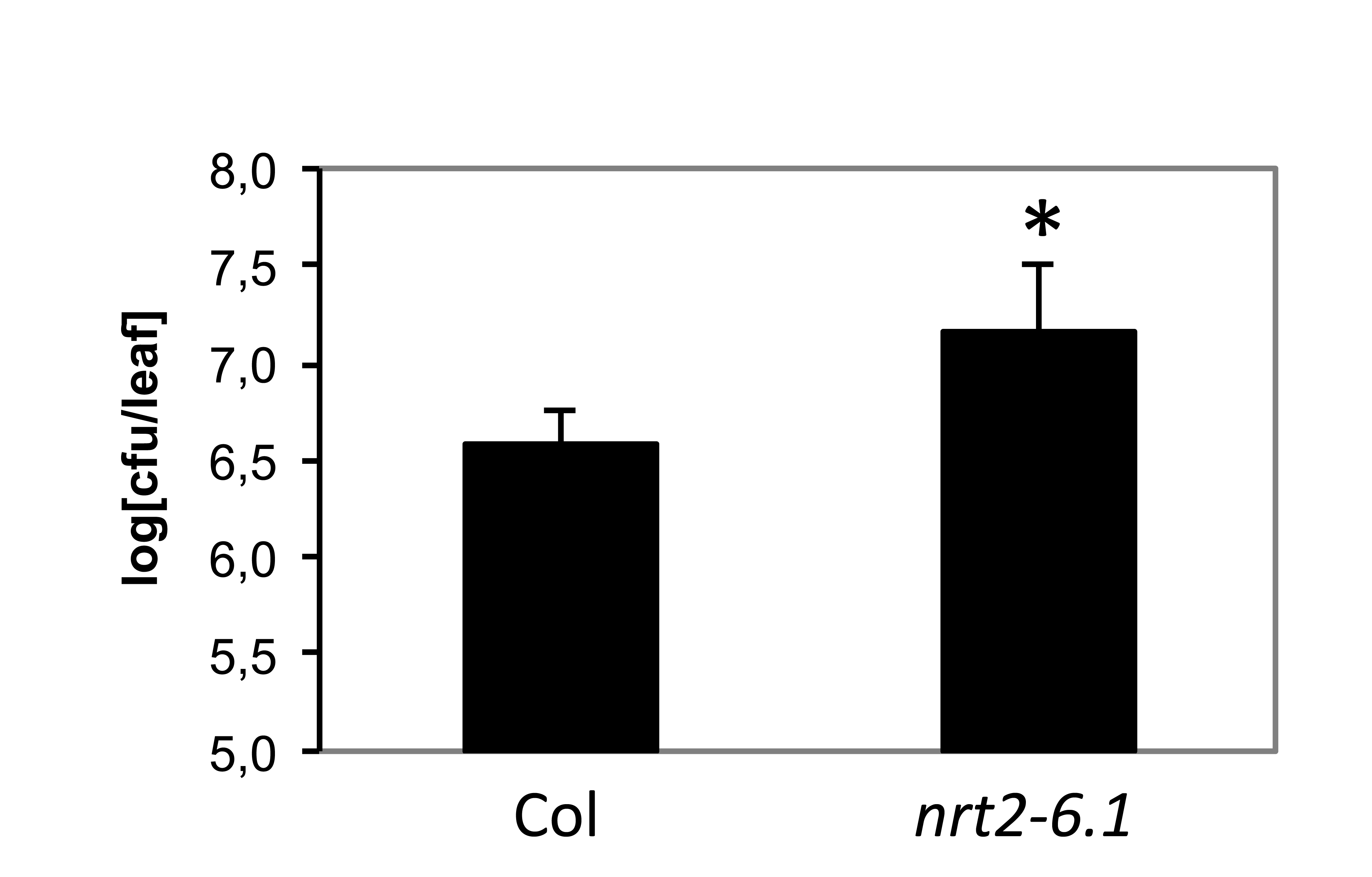

Supplement: Figure S4 — The nrt2.6-1 mutant supports higher bacterial multiplication of E. amylovora cells than wild-type plants. Bacterial count of E. amylovora in wild-type (Col) and mutant (nrt2.6-1) plants. The number of CFU present in leaf extracts was counted 24 h post inoculation. The asterisk indicates that the means are statistically different according to Mann and Whitney’s test (P value <0.05). (TIF) [file pone.0042491.s004.tif]

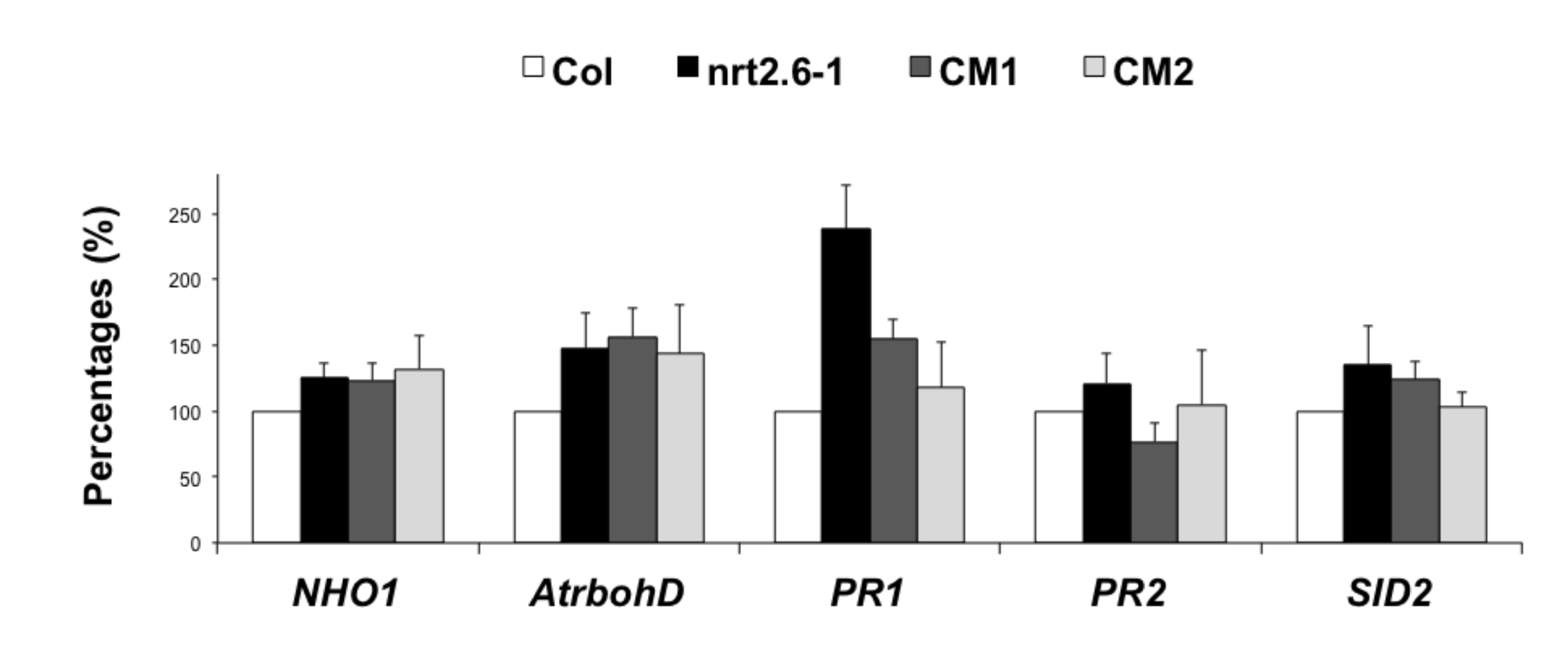

Supplement: Figure S5 — Defense gene expression in response to infection by E. amylovora . Expression of marker genes was measured 24 h after E. amylovora inoculation. A 100% arbitrary value was affected to expression levels in Col. (TIF) [file pone.0042491.s005.tif]

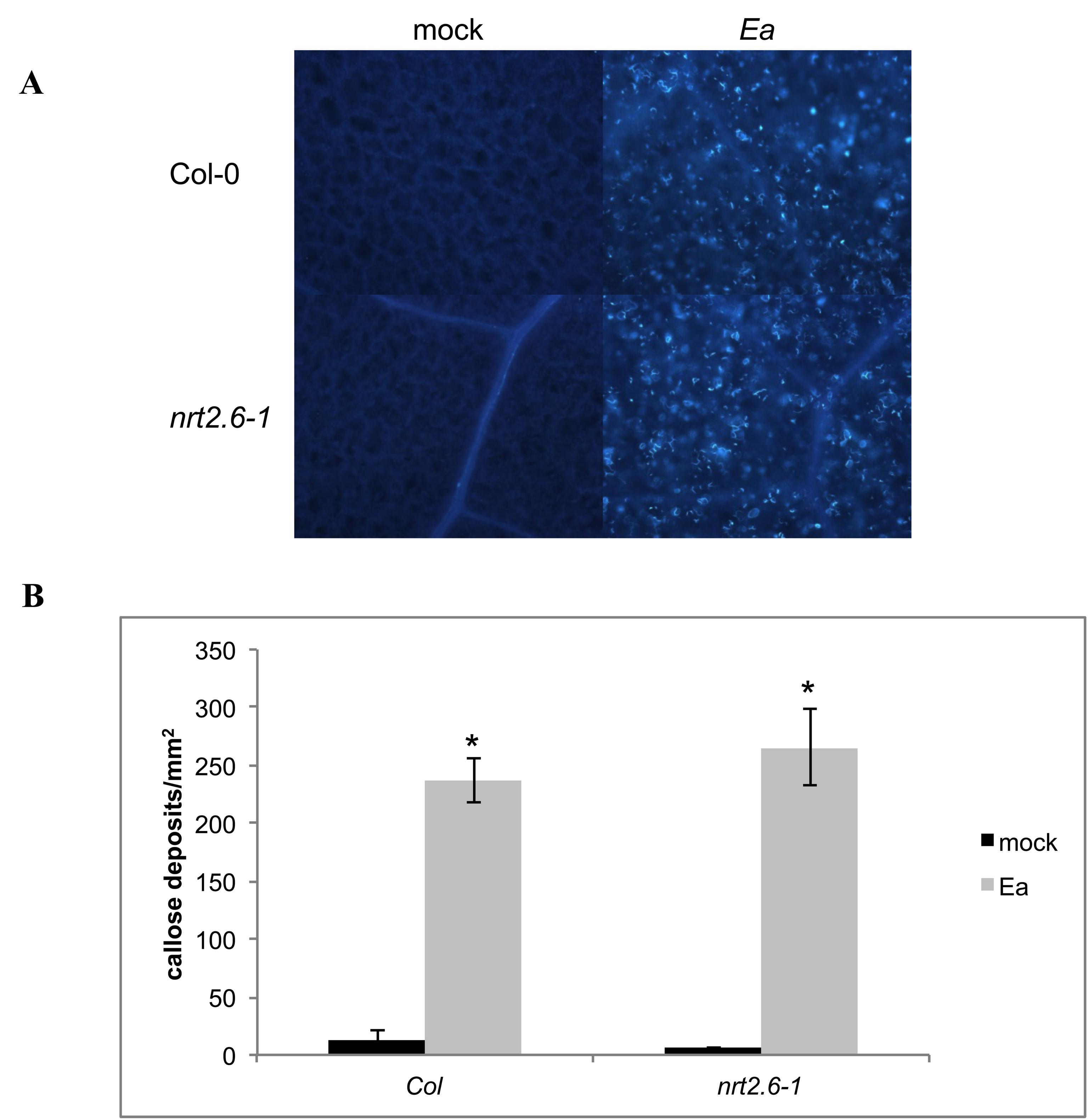

Supplement: Figure S6 — Callose accumulation in response to E. amylovora is not affected in the nrt2.6-1 mutant. Analysis of callose deposits in E. amylovora-inoculated wild-type (Col) and mutant (nrt2.6-1) plants. Leaves were collected 8 hpi and stained with aniline blue as described previously [31]. No significant difference in callose deposition could be observed between wild-type and mutant plants. A: Representative images are shown for each treatment. B: Experiments were repeated twice with similar results. The asterisks indicate that the means are statistically different between mock and Ea treatments according to Mann and Whitney’s test (P value <0.05). No statistical differences were found between wild-type and nrt2.6-1 mutant. (TIF) [file pone.0042491.s006.tif]

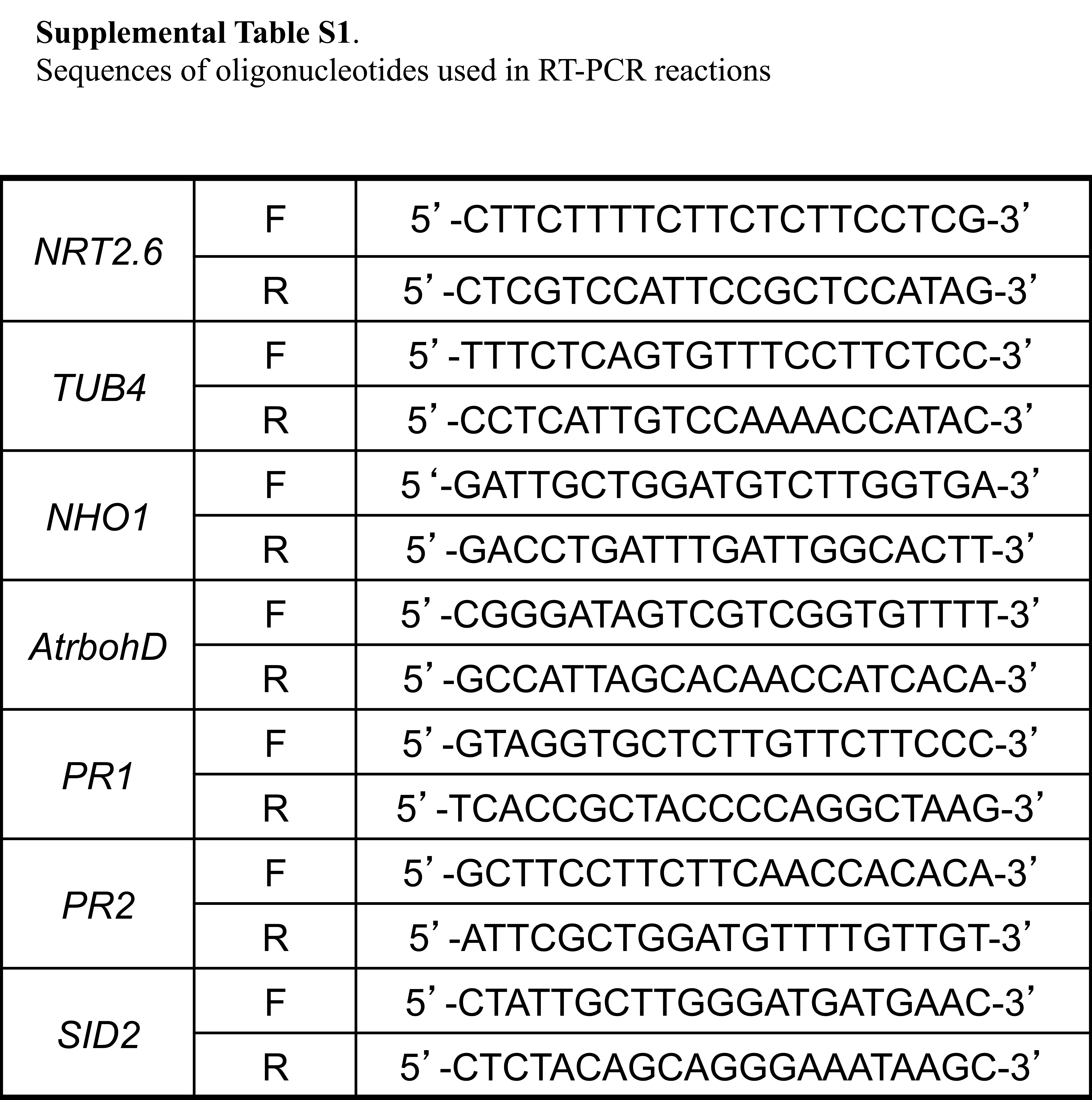

Supplement: Table S1 — Sequences of oligonucleotides used in RT-PCR reactions. This table summarizes the sequences of oligonucleotides used in this study. For each targeted gene, the sequences of forward (F) primer and reverse (R) primer are given. (TIF) [file pone.0042491.s007.tif]
